# Supplementary material for: Individual and community-level determinants of skilled birth attendant delivery in Ethiopia; multilevel analysis
Source: PLoS One. 2023 Aug 1;18(8):e0289145. doi: 10.1371/journal.pone.0289145 (PMC10393148; doi:10.1371/journal.pone.0289145)
Supplement: Attachment — Submitted filename: Response to Reviewers.docx [file pone.0289145.s002.docx]

**Rebuttal letter	Date, February 2, 2023**

**Subject; submission of revised manuscript (PONE-D-22-07743)**

**Individual and Community-level Determinants of Skilled Birth Attendant Delivery in Ethiopia; Multilevel Analysis**

**Hiwotie Getaneh Ayalew**

**To PLOS ONE**

Dear all,

We would like to thank you for these constructive, building, and improvable comments on this manuscript that would improve the substance and content of the manuscript. We considered each comment and clarification question of editors and reviewers on the manuscript thoroughly. Our point-by-point responses for each comment and question are described in detail on the following pages. Further, the details of changes were shown by track changes in the supplementary document attached. The manuscript language was further improved in the revised manuscript. and we follow journal guidelines. We have also revised the statistical analysis method again and we have attached recent comments in a point-by-point response.

**Version 1; editor’s comments**

1. Please ensure that your manuscript meets PLOS ONE's style requirements, including those for file naming.

**Authors’ response**; thank you dear editor we have prepared the documents based on PLOS ONE requirements.

2. Thank you for stating the following financial disclosure: “no”

At this time, please address the following queries:

a)        Please clarify the sources of funding (financial or material support) for your study. List the grants or organizations that supported your study, including funding received from your institution.

b)        State what role the funders took in the study. If the funders had no role in your study, please state: “The funders had no role in study design, data collection and analysis, decision to publish, or preparation of the manuscript.”

c)        If any authors received a salary from any of your funders, please state which authors and which funders.

d)        If you did not receive any funding for this study, please state: “The authors received no specific funding for this work.”

**Authors’ response;** thanks, dear editor. We have addressed this comment by stating that “The authors received no specific funding for this work.”

3. Thank you for stating the following in your Competing Interests section. “NO” Please complete your Competing Interests on the online submission form to state any Competing Interests. If you have no competing interests, please state "The authors have declared that no competing interests exist.", as detailed online in our guide for authors at <http://journals.plos.org/plosone/s/submit-now>

**Authors’ response;** thanks, dear editor. We have explained the competing interest as “The authors have declared that no competing interests exist” both in the manuscript and in the online submission section.

**Version 2; reviewers’ comments**

1. Is the manuscript technically sound, and do the data support the conclusions?
The manuscript must describe a technically sound piece of scientific research with data that supports the conclusions. Experiments must have been conducted rigorously, with appropriate controls, replication, and sample sizes. The conclusions must be drawn appropriately based on the data presented.

Reviewer #1: Partly

Reviewer #2: Yes

**Authors’ response**; thank you, dear reviewers. We have improved the revised version of the manuscript.

2. Has the statistical analysis been performed appropriately and rigorously?

Reviewer #1: Yes

Reviewer #2: Yes

**Authors’ response**; thank you, dear reviewers.

3. Have the authors made all data underlying the findings in their manuscript fully available?
The [PLOS Data policy](http://www.plosone.org/static/policies.action#sharing) requires authors to make all data underlying the findings described in their manuscript fully available without restriction, with rare exception (please refer to the Data Availability Statement in the manuscript PDF file). The data should be provided as part of the manuscript or its supporting information, or deposited to a public repository. For example, in addition to summary statistics, the data points behind means, medians and variance measures should be available. If there are restrictions on publicly sharing data—e.g. participant privacy or use of data from a third party—those must be specified.

Reviewer #1: Yes

Reviewer #2: Yes

**Authors’ response**; Thank you, dear reviewers. We have stated that the data was fully available without restriction in the revised manuscript at the declaration session and online submission.

4. Is the manuscript presented in an intelligible fashion and written in Standard English?
PLOS ONE does not copyedit accepted manuscripts, so the language in submitted articles must be clear, correct, and unambiguous. Any typographical or grammatical errors should be corrected at revision, so please note any specific errors here.

Reviewer #1: No

Reviewer #2: Yes

**Authors’ response**; thanks a lot, dear reviewer. We have critically improved the readability of the manuscript. Please see the revised version.

**Reviewer 1 Comments**

**Comments #1.** There are numerous typographical and grammatical problems throughout the document which need thorough revision.

**Authors’ response;** Thanks a lot dear reviewer for your critical comment to improve the manuscript. We have fully accepted your comment and we have revised the manuscript to reduce the typological and grammatical errors.

**Comment #2.** In the Introduction, additional justification is needed in the introduction section to justify the novelty of the study, “Even though skilled birth attendant delivery depends on both individual and community-level determinants, still, limited studies have been done beyond individual-level factors” is not enough of a justification since numerous significant factors this study reported had also been reported by other similar studies (which are used in the present study as a reference also)
**Authors’ response**; thanks a lot dear reviewer for your unlimited effort to improve the manuscript. We accepted your comment and included sound justification in the revised manuscript.
**Comment #3**. In the Method section, It is important to specify whether the independent variables (individual and community) were taken as they are or were classified by the authors, for instance, was PCA conducted for the wealth Index?

**Authors’ response;** thanks, dear reviewer for your detailed revision. We have used some variables as coded as DHS data set and some variables which do not fulfill the chi-square assumption were recoded again based on some previous studies. Regarding the wealth index, PCA was conducted by the DHS program data analyzer. Dear reviewer, we kindly request you to see the DHS recode manual guide, which further expressed, how PCA was done for the wealth index.

**Comment #4**. It is also important to include the measurement section in the Method for some of the variables especially for Community poverty, Community media exposure---

**Authors’ response;** Thanks a lot, dear reviewer. We have expressed in the revised manuscript, how these community-level factors are measured. we kindly request you see the revised manuscript.

**Comment #5**. The writing especially in the result section could be improved, what is written is stand-alone sentences, try connecting using conjunctions during interpretations. Additionally, the grammar needs improvements, use writing applications.

**Authors’ response;** Thank you in advance dear reviewer. We have tried to revise the result section in the revised manuscript. We have also used Grammarly and expert persons in English for the improvement of the revised manuscript.

**Comment #6**. The discussion section is poor and limited in justifying the similarity and differences with other studies using scientific facts. References (scientific facts) should be used to explain and back claims of significant association, similarities, and differences with other studies. The entirety of the discussion needs more reflective writing and synthesis
**Authors’ response;** Thank you dear reviewer for your great effort to review our manuscript. We have revised the discussion again and added some scientific evidence with their references in the revised manuscript.

**Comment #7**. The citations need to be consistent all over, for instance, in the sentence, “This finding was supported by studies done in Ethiopia (31).” The ‘studies’ were not cited in the manuscript

**Authors’ response**; Thanks dear reviewer we have appreciated your comments. We have revised the typing error. we kindly request you see it in the revised manuscript.

**Comment #8**. The final paragraph of the discussion deals with the Strengths and Limitations of the study, which I would prefer as an independent section but it is perfectly fine to include under discussion also.

**Authors’ response**; Thanks a lot dear reviewer for your deep comments.

**Comment #9.** The Acronym and abbreviation section is not consistent with the document, limit to those used three or more times in the manuscript, have you even used RR?. Are they abbreviations or acronyms?

**Authors’ response;** thank you, dear reviewer, for your unlimited effort. The abbreviation RR was not used and we remove it in the revised manuscript.

**Comment #10.** Rewrite the acknowledgment so that it could make grammatical sense, Measure DHS? Also, please ensure that, before resubmission, a person proficient in written English edits the manuscript. It is important that the message being conveyed in the manuscript is as unambiguous as possible

**Authors’ response;** thanks dear reviewer for your constructive comments. We have revised and rewritten the acknowledgment in the revised manuscript. Dear reviewer a person proficient in the English language revised and edits the revised manuscript before submission.

**Reviewer #2 comments**

The study aims to assess the individual and community-level factors associated with Skilled Birth Attendant (SBA) delivery in Ethiopia.

**Comment #1.** **Abstracts**

The full name for ANC is to be stated prior to the use of the abbreviation ANC

**Authors’ response;** thanks, dear reviewer. We have accepted your comment. We have stated ANC before using it as an abbreviation in the revised manuscript.

**Comment #2. Method**

Statement 'Finally, AOR with 95% CI and random effects were reported' requires revision..

**Authors’ response;** thank your dear reviewer for your contractive comment

**Comment# 3. Results**

For 'wealth index(AOR=0.64;95% CI 0.46-0.87)' the figures to be spaced out

**Authors’ response;** thank you, dear reviewer. we have edited the revised manuscript.

**Comment #4. Introduction**

Paragraph 1, skilled birth attended. The words attendance or attended or attendants are to be standardized where necessary throughout the manuscript.
Paragraph 3, typo error ‘SBAdelivery’. The subsequent words ‘skilled birth attendant’ is to be replaced with SBA.

**Authors’ response;** thank you, dear reviewer. We strongly appreciate your comment. We have revised the introduction in the revised manuscript and we take consistent words for the skilled birth attendant. We also replaced skilled birth attendant delivery with SBA delivery.

**Comment #5. Independent variables**

Typo error ‘visit(No visit’

**Authors’ response;** Thanks a lot, dear reviewer. We have edited the type error.
**Comment #6. Multilevel logistic regression analysis**

Typo error ‘ regression model Variables which’
Statement ‘Bivariable multilevel logistic regression analysis were considered for the individual and community level model adjustments for the multivariable multilevel logistic regression model’ requires revision.
Typo error ‘log likely hood’, ‘highest log likely hood ratio’
Statement ‘Finally, the adjusted odds ratio with a 95% confidence interval was reported for statistically significant variables.’ requires revision.
A statement on multicollinearity assessment (if any) to be included.

**Authors’ response;** thanks dear reviewer we appreciate your comments. We have edited the suggested typing errors in the revised manuscript and we have revised the statements again. Regarding multicollinearity assessment,we have again revised data set extracted for analysis and cheked multicolinerity for indipendant variables. Those variables with VIF less than 10 were taken to fit the model.

**Comment #7.Results**

Random effect analysis results, Technically p value cannot be zero (to use symbol p<)
Fixed effect analysis results,

For the statement ‘Women who started ANC visit first trimester and second trimester had 1.87(AOR=1.87:95%CI 1.09-3.20) and 2.65 (AOR=2.65;95% CI 1.52-4.65)’ the figures 1.87(AOR=1.87:95%CI 1.09-3.20) and 2.65 (AOR=2.65;95% CI 1.52-4.65) are to be referred as second and first trimester respectively in the text.

AIC and BIC are to be included as part of the model fit diagnostic. **Authors’ response;** thanks a lot dear reviewer we have critically improved typing and technical errors. Regarding model comparison for nested model as you know devieance and loglikelihood ratio are commonly used and recommended since they test a hypothesis that weather additional parameter has improved model fit or not. Whereas AIC and BIC are commonly used to compare models from different families with lowest value being best. Therefore, in our analysis multilevel model is hierarchical and models are nested (Singer J, Willett J, Singer J, Willett J. Doing data analysis with the multilevel model for change. Applied longitudinal data analysis: Modeling change and event occurrence. 2003:96-7.). On the other hand,Akaike Information Criterion (Akaike,1974) and Bayesian Information Criterion (schwarz,1978) are a measure of goodness of fit for model summaries. These are penalized for many parameters. A smaller value is better, but no absolute good value, whereas loglikelihood hood and deviance are unpenalized goodness of fit. Always negative, larger (close to zero) is better .deviance is -2log likelihood and the smaller is better. Hence we preferred to use log-likelihood and deviance for the model fitness measure .

**Comment#8 Discussion**
References to be spaced out e.g. East Africa(10), Ghana(14), and East Africa(10).

**Authors’ response;** thanks, dear reviewer. We have edited the typing error.

**Comment #9 Conclusion**
Typo ‘It is Better to

**Authors’ response;** thanks in advance, dear reviewer

**Thanks, a lot!!!**
