# Supplementary material for: Individual and community-level determinants of skilled birth attendant delivery in Ethiopia; multilevel analysis
Source: PLoS One. 2023 Aug 1;18(8):e0289145. doi: 10.1371/journal.pone.0289145 (PMC10393148; doi:10.1371/journal.pone.0289145)
Supplement: Attachment — Submitted filename: Response to Reviewers.docx [file pone.0289145.s004.docx]

**Rebuttal letter	May 17, 2023**

**Subject; submission of revised manuscript (PONE-D-22-07743)**

**INDIVIDUAL AND COMMUNITY-LEVEL DETERMINANTS OF SKILLED BIRTH ATTENDANT DELIVERY IN ETHIOPIA; MULTILEVEL ANALYSIS**

**Hiwotie Getaneh Ayalew**

**To PLOS ONE**

Dear all,

We would like to thank you for these constructive, building, and improvable comments on this manuscript that would improve the substance and content of the manuscript. We considered each comment and clarification question of editors and reviewers on the manuscript thoroughly. Our point-by-point responses for each comment and question are described in detail on the following pages. Further, the details of changes were shown by track changes in the supplementary document attached.

**editor’s comments**

Dear Dr. Ayalew,

Please review your reference list to ensure that it is complete and correct. If you have cited papers that have been retracted, please include the rationale for doing so in the manuscript text, or remove these references and replace them with relevant current references. Any changes to the reference list should be mentioned in the rebuttal letter that accompanies your revised manuscript. If you need to cite a retracted article, indicate the article’s retracted status in the References list and also include a citation and full reference for the retraction notice.

**Authors’ response:** many thanks dear reviewer for giving me additional opportunity to revise the manuscript. I have checked all the references and they are correct and complete. No reteracted articles are cited.

**Comment 1:** Under your recommendation (in both abstract and recommendation section) you have recommended, increasing the timely initiation of ANC through health education. But, what evidence do you have as the gap is the gap of knowledge among the women?? how do you justify your recommendation from the perspective of what the government is currently doing?
your response is not inline with the question. Recommendation should be inline with your finding. But, what you present as an evidence is already existing knowledge, not your finding

**Authors’ response:** many thanks dear editor. We are much convinced with your suggestion and we revised the recommendation section in the abstract and main text (kindly refer too revised manuscript.

**Comment 2**: you have explained as the community level factors were grouped based on median because of the skewness of the data. the question here is since the community level variables are categorical, how did you check for normality??? is it possible? Authors response; thank you, dear editor. yes, it is possible. we have used a histogram to check the normality of the data for analysis. its miracle for me to use histogram for categorical data, which is totally impossible. please, read biostatistics on data presentation

**Authors’ response:** thank you so much dear reviewer. Sorry for creating confusion. Ofcurse as you said it is imposible to check normality by using histogram for categorical variable. Let me explain again how we come across community level variables. Initially these ggregated community level variables are proportions not a categorical variables. For instance, community media exposure is a proportion computed by dividing number of individuals exposed to media to total population in that cluster. As you know proportion is continuous variable and to categorize as high and low media exposure level we need to use either mean (if the proportion is normally distributed) or median (if the proportion is skewed). To decide weither mean or mrdian should be used for categorizing proportion of media exposure we ckecked normality by using histogram and skewed distribution was observed. Then we used median for categorization. It is therefore, for the proportion that histogram is used not for categorized variable. A similar approach was used for community poverty level and we have clearly stated these in the method section and we kindly refer to pragraph six of method section. We believe we have clarified it now.

Many thanks for your commitment to improve the quality of our manuscript.
