# Supplementary material for: Individual and community-level determinants of skilled birth attendant delivery in Ethiopia; multilevel analysis
Source: PLoS One. 2023 Aug 1;18(8):e0289145. doi: 10.1371/journal.pone.0289145 (PMC10393148; doi:10.1371/journal.pone.0289145)
Supplement: Attachment — Submitted filename: Reviewer comments docx.docx [file pone.0289145.s001.docx]

**Reviewer Comments**

#1. There are numerous typographical and grammatical problems throughout the document which need thorough revision

#2. In the Introduction, Additional justification is needed in the introduction section to justify the novelty of the study, *“Even though skilled birth attendant delivery depends on both individual and community-level determinants, still, limited studies have been done beyond individual-level factors”* is not enough of a justification since numerous significant factors this study reported had also been reported by other similar studies (which are used in the present study as a reference also)

#3. In the Method section, It is important to specify whether the independent variables (individual and community) were taken as they are or were classified by the authors, for instance, was PCA conducted for the wealth Index?

#4. It is also important to include the measurement section in the Method for some of the variables especially for Community poverty, Community media exposure---

#5. The writing especially in the result section could be improved, what is written is stand-alone sentences, try connecting using conjunctions during interpretations. Additionally, the grammar needs improvements, use writing applications.

#6. The discussion section is poor and limited in justifying the similarity and differences with other studies using scientific facts. References (scientific facts) should be used to explain and back claims of significant association, similarities, and differences with other studies. The entirety of the discussion needs more reflective writing and synthesis.

#7. The citations need to be consistent all over, for instance, in the sentence, “*This finding was supported by studies done in Ethiopia (31).”* The ‘studies’ were not cited in the manuscript

#8. The final paragraph of the discussion deals with the Strengths and Limitations of the study, which I would prefer as an independent section but it is perfectly fine to include under discussion also.

#9. The Acronym and abbreviation section is not consistent with the document, limit to those used three or more times in the manuscript, have you even used RR?. Are they abbreviations or acronyms?

#10. Rewrite the acknowledgment so that it could make grammatical sense, Measure DHS?

Also, please ensure that, before resubmission, a person proficient in written English edits the manuscript. It is important that the message being conveyed in the manuscript is as unambiguous as possible
